# Hematophagous Endeavors, Fact and Fancy

**DOI:** 10.3201/eid2308.AC2308

**Published:** 2017-08

**Authors:** Byron Breedlove, Paul M. Arguin

**Affiliations:** Centers for Disease Control and Prevention, Atlanta, Georgia, USA

**Keywords:** art science connection, emerging infectious diseases, art and medicine, about the cover, Alexander Skachkov, Hematophagous Endeavors, Fact and Fancy, Old Mosquito, mosquitoes, mosquito-borne diseases, malaria, parasites, public health

**Figure:**
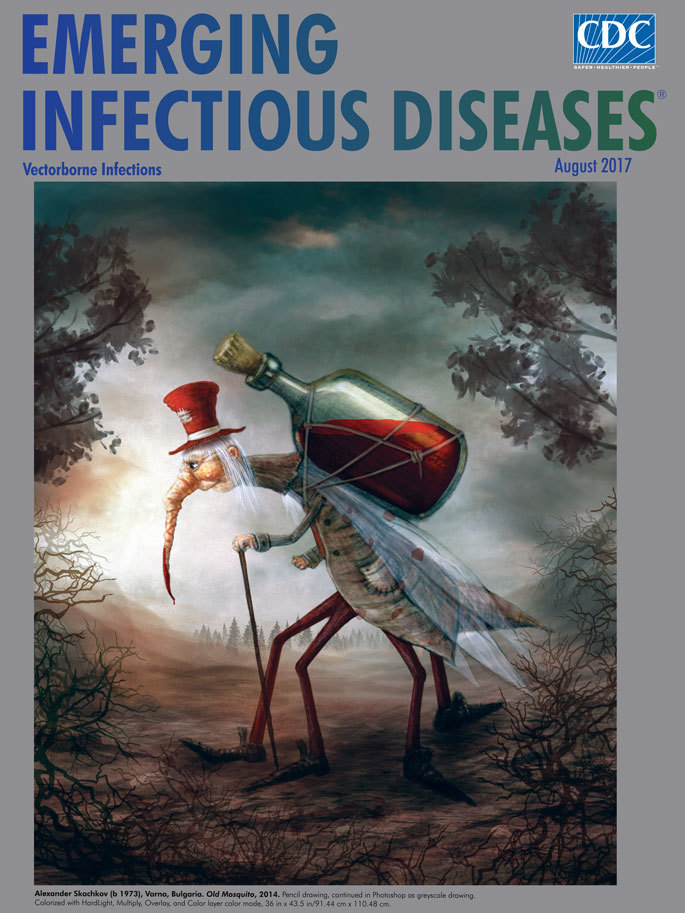
**Alexander Skachkov (b 1973), Varna, Bulgaria. *Old Mosquito*, 2014**. Pencil drawing, continued in Photoshop as grayscale drawing. Colorized with Hard Light, Multiply, Overlay, and Color layer color mode, 36 in × 43.5 in/91.44 cm × 110.48 cm.

Let’s start with some facts. The word “mosquito” is Spanish for “little fly.” The single family Culicidae comprises more than 3,500 species of mosquitoes, and these ectoparasites are found in a wide range of environments spanning the globe with the exception of Antarctica. The life span of adult mosquitoes ranges from 2 weeks to 6 months.

Mosquitoes belonging to about three quarters of recognized species consume blood. Female mosquitoes of those species are equipped with tubular mouthparts that can pierce the skin of their human and animal hosts to consume blood. The blood provides them with protein to produce eggs. When they are not producing eggs, female mosquitoes typically consume the same things that males do, nectar and sap from a variety of plants. Male mosquitoes do not need to feed on blood and consequently have not evolved to have larger mandibles for piercing layers of skin.

Some mosquitoes spread disease-causing agents that have serious and widespread consequences for humans and animals. Mosquitoes transmit the five *Plasmodium* parasite species that cause malaria in humans and the infectious agents that can cause chikungunya disease, dengue hemorrhagic fever, Japanese encephalitis, lymphatic filariasis, Rift Valley fever, West Nile virus infection, and yellow fever. Of those, the illness that sickens and kills most people each year is malaria. For the year 2015, the World Health Organization reported 212 million new cases of malaria and an estimated 429,000 deaths from malaria worldwide.

Mosquitoes are not simply mechanical vectors or mobile fomites. Many pathogens complete stages of their life cycles within the mosquito or may have to move from the mosquito’s gut to its salivary glands—which is often why mosquitoes are not immediately infectious after consuming a blood meal from the initial infectious host. This complex relationship helps explain why specific diseases and certain mosquitoes are linked; malaria and *Anopheles* spp., Japanese encephalitis and *Culex* spp., or dengue and *Aedes* spp.

Bill Gates wrote, “When it comes to killing humans, no other animal even comes close.” Science writer Jerry Adler noted in an article, “One species, the *Anopheles gambiae* mosquito, has been called the world’s most dangerous animal, although strictly speaking that applies only to the female of the species, which does the bloodsucking and harms only indirectly.”

As we consider the facts, let’s shift to fancy for this month’s cover art, *Old Mosquito,* by artist, illustrator, and web designer Alexander Skachkov. Much of his art belongs to realms of magic, whimsy, and wonder; his colorful creations often feature a wry sense of humor. He cites as inspiration the contemporary fantasy art created by Scott Gustafson, Paul Bonner, and Jean-Baptiste Monge. Art blogger Lafayette Wattles states that Skachkov “offers a fascinating mix of nature and humanity with neither being quite what we’ve come to expect in the real world.”

Although Skachkov is not working with bristle brushes and a palette of paints, his approach is nonetheless laborious and deliberate. Skachkov’s creations, including this work, typically start with a penciled sketch that forms the basis of the finished image. He scans that drawing into Photoshop, with which he employs a range of tools, filters, layers, and effects to manipulate the textures, tones, and colors.

In this clever image, Skachkov depicts a tired older mosquito heading home after a long night of collecting blood. The bare branches and grayish fog of morning suggest that summer is past and the old mosquito is approaching the end of his days in the end of the year. The bright red blood contained in the mosquito’s jug, his red hat and legs, and, of course, his blood-tipped proboscis tinged from its hematophagous endeavors, contrasts with the morning gloom.

Entomologists will be quick to point out that the depiction is scientifically inaccurate because male mosquitoes do not ingest blood. Mosquitoes also do not wear shoes and cute hats (so we hope any entomologists reading this essay will allow the illustrator to exercise artistic license). Skachkov has depicted this recent blood collection not just as the mosquito’s prized possession, but also as his burden. The mosquito is bowed with the weight of the bottle strapped on his back. Supported by a cane, he trudges along collecting blood in exchange for an itchy welt or worse—an infectious microorganism that can cause illness, disability, and death for its new host. His resigned expression suggests that our mosquito must be aware of the grim consequences of his actions but has no choice regarding his role in the world. With few friends and not much to smile about, this older mosquito will continue on his appointed rounds for as long as he can bear it.
